# Health behaviors of late adolescents in China: Scale development and preliminary validation

**DOI:** 10.3389/fpsyg.2022.1004364

**Published:** 2022-11-11

**Authors:** Qian Qiu, Shengting Dai, Jingfei Yan

**Affiliations:** ^1^Key Laboratory of Adolescent Health Assessment and Exercise Intervention of Ministry of Education, East China Normal University, Shanghai, China; ^2^College of Physical Education and Health, East China Normal University, Shanghai, China; ^3^School of Sports Science and Engineering, East China University of Science and Technology, Shanghai, China

**Keywords:** health behavior, late adolescents, scale development, factor analysis, construct validity

## Abstract

Health behaviors influence health and well-being, improve quality of life, and provide economic benefits. It is important to take advantage of health-related opportunities during adolescence. Staying healthy during adolescence also promotes the future well-being of individuals and that of the next generation. We aimed to develop a reliable and valid scale based on the General Senior High School Physical Education and Health Curriculum Standards (2017 edition) to evaluate Chinese late adolescents’ health behavior. The scale was to help physical education teachers measure the health behavior level of senior high school students, improve physical education and health teaching, and promote Chinese adolescent health. Participants were recruited by convenience sampling from September to October 2019. For the first survey, we recruited 526 senior high school students (318 boys, 208 girls; M_age_ = 16.5), and the data were subjected to item analysis and exploratory factor analysis. For the second survey, we recruited 542 senior high school students (249 boys, 293 girls; M_age_ = 15.5), and the data were subjected to confirmatory factor analysis and internal consistency reliability analysis. After exploratory factor analysis, we extracted four factors with 23 items: exercise awareness and habits (five items), mastering and applying healthy behavior knowledge (10 items), emotional regulation (four items), and environment adaptation (four items). The Cronbach’s alpha values for these factors ranged from 0.863 to 0.937. After confirmatory factor analysis, we achieved a satisfactory goodness-of-fit model (CMIN/DF = 2.92, RMR = 0.03, GFI = 0.93, CFI = 0.91, TLI = 0.92, RMSEA = 0.06). Internal consistency, test–retest reliability, and construct validity were all satisfactory. These results suggest that the Chinese version of the Health Behavior Scale is a reliable and valid instrument for assessing the health behavior of senior high school students. The findings have important implications for increasing adolescents’ health literacy, promoting adolescents’ health, and enhancing the well-being of late adolescents.

## Introduction

Health behaviors broadly refer to actions taken by individuals that affect health, disease, and mortality (Short and Mollborn, 2015; Rubinelli and Diviani, 2020). Healthy behavior such as physical activity, a reasonable diet, not smoking, and not being addicted to alcohol could decrease the risk of chronic diseases (i.e., obesity, cardiovascular disease, and cancer), improve quality of life, and provide substantial economic benefits (Crone et al., 2019; Bilal et al., 2020; Hecht et al., 2020; Stephanie et al., 2020). The adolescent years are a critical transitional period during which rapid physical, emotional, cognitive, and social development occurs (Shlafer et al., 2014; Inchley et al., 2020; Roberts et al., 2020). Health behavior in the early stages of life has an impact on health consequences in later life (Umberson et al., 2010; Akasaki et al., 2019). Studies have found that many of the major behavioral risk factors which lead to non-communicable diseases (smoking, drinking, and sedentary lifestyle) are mainly formed during adolescence and affect habit formation well into adulthood (Inchley et al., 2020).

Moreover, the latter phase of adolescent brain development (15–19 years) includes the continued development of executive and self-regulatory skills, leading to a greater future orientation and an increased ability to weigh the short-term and long-term implications of decisions (Patton et al., 2016). Therefore, it is particularly important to cultivate and evaluate late adolescent health behaviors. Previous research has indicated that many adult health behaviors are developed and established during late adolescence and early adulthood (Liu et al., 2019). Behaviors developed during adolescence frequently persist into late adulthood (Lien et al., 2002; Berge et al., 2015), which is a transitional period during which adolescents experience physical, mental, and social development transformations. Thus, it is an important period for social and cognitive development. These years also lay the groundwork for a successful transition into a healthy and independent lifestyle and employment, and provide support for life partnerships, marriage, and parenthood (World Bank, 2006; Patton et al., 2016).

Several international organizations such as Health Behaviour in School-aged Children (HBSC) and Global Action for Measurement of Adolescent health (GAMA) are dedicated to gaining insight into adolescent health by measuring health behavior to make effective, efficient, and accountable investments (Azzopardi et al., 2017). An important milestone of international health behavior research, the HBSC study, in collaboration with the WHO Regional Office for Europe, has been conducted every 4 years in 50 countries across Europe and North America since 1982, aiming to inform policy and practices to improve the lives of millions of young people (Health Behaviour in School-aged Children, 2001). GAMA, which defined a core set of adolescent health indicators, was established by the WHO and UN partner agencies in 2017 and aimed to unify efforts toward adolescent health measurement and reporting (World Health Organization, 2017). The Health Promotion Lifestyle Profile (HPLP), a well-known instrument measuring health promotion lifestyle behaviors, was originally developed by Walker in 1987 and revised as HPLP-II in 1995 (Walker et al., 1995). The HPLP includes 48 items on six subscales, and the HPLP-II includes 52 items on six subscales: health responsibility, physical activity, nutrition, interpersonal relations, spiritual growth, and stress management. The HPLP have been applied in many countries and different populations, with good reliability and validity. However, several shortcomings of the current approaches for evaluating adolescent health were identified: First, more qualitative research is needed regarding mental health, injury, and positive measures of adolescent health and well-being. Second, the link between global and national indicators, as well as between indicators and programming at national and subnational levels is often missing (Guthold et al., 2019). Third, most of the existing instruments for assessing behavioral outcomes, measure the duration, frequency, and time of occurrence of health behavior. Few studies have measured the adolescent’s health behavior perceived ability, such as the acquisition and application of knowledge about health behaviors, especially after that the DeSeCo Projects conceptual framework for key competencies. In China, measurements of health behaviors include studies using the revised version of the internationally validated scales, as well as self-developed scales for specific behavior or disease. But the existing scales inability to assess the consciousness and ability of emotion regulation and environmental adaption and exercise. No comprehensive measurement tool for health behavior. There are few synthetic data sources on adolescent health behavior in this discipline. There are few synthetic data sources on adolescent health behavior in this discipline.

In China, the physical education and health curriculum is undergoing significant reform, and the definition of health behavior has also changed. Health behavior became one of the course objectives of the Chinese senior high school physical education and health curriculum, in 2018. The Ministry of Education of the People’s Republic of China published the General Senior High School Physical Education and Health Curriculum Standards (2017 edition), which put forward the concept of core literacy in physical education and health (The Ministry of Education of the People's Republic of China, 2018). The national curriculum standard indicated sports ability, health behavior, and sports ethics as the main components of core literacy in the physical education and health discipline. The curriculum standard explicitly stated definitions, content, and classifications (Liu, 2018; Liu and Bing Shu, 2018). Exercise awareness and habits, mastering and applying healthy behavior knowledge, emotional regulation, and environment adaptation were the four major parts of health behavior. To the best of our knowledge, there is little research and almost no existing validated health behavior scales based on the standard curriculum (2017 edition), and few studies combined the health behavior traits. Therefore, the purpose of this study was to develop a reliable and valid scale to evaluate the health behaviors of senior high school students based on the viewpoint of health behavior proposed in the General Senior High School Physical Education and Health Curriculum Standards (2017 edition). The Health Behavior Scale was to help physical education teachers measure the health behavior level of senior high school students, improve physical education and health teaching, and promote Chinese adolescent health and well-being.

## Materials and methods

### Original items for scale development

To evaluation of senior high school students’ health behaviors, we first analyzed the documents and literature about health behavior and physical literacy before March 2019. Then developing the interview guide for the interview discussion (Appendix 1) and creating the original items pool through group discussion. Three group discussion sessions were conducted, with each lasting for an average of 90 min. The interviews were conducted utilizing both open- and closed-ended questions. The participants consisted of physical education subject specialists (N = 5) and physical education teachers (N = 5) in China. Analyze the interview results through group discussion, delete the repeated content, and add the new content to the item pool. In the group discussion sessions, participants shared their perception of health behavior based on the curriculum standard (2017 version), analyzed the structure of the scale, evaluated the item pool, and gave suggestions for revision (Appendix 2). The discussion sessions were audio-recorded and supplemented by hand-written notes.

The third revision of the scale, namely the preliminary scale, was then sent to a panel of four experts who were teaching and conducting research in the area of physical education and health education. They were invited to evaluate the face validity of the preliminary scale. The experts did not suggest the addition or deletion of any of the items, and the preliminary scale was well received and praised as useful.

Four sources were used for the evaluation of senior high school students’ health behaviors. First, related items were compiled from the content of physical education and health textbooks for senior high school students approved by the Ministry of Education and physical education and fitness textbooks published by Shanghai Education Press. Second, items were searched for in literature related to health behaviors in senior high school students. Third, entries on the characteristics of senior high school students were compiled and combined with the current background. Fourth, other items referenced related to health behavior were also included.

### Participants

We adopted convenience sampling, recruiting students from public schools that could provide a representative population for this study due to the school size being representative of most senior high schools. Calculation of the sample size was performed according to the criteria established by Kline which recommend a ratio of 5–10 subjects per item (Kline, 2010). Thus, the sizes of our samples in the process of item selection and reliability and validity analysis were decided based on this rule. Two surveys were conducted in Shanghai, China. The two surveys used two health behavior scales that covered four aspects of health behavior. The first survey is provided in Appendix 3, completed in September 2019. The second survey is provided in Appendix 4, completed in October 2019. Sample 1, on whom the preliminary scale was conducted, comprised 526 senior high school students (318 boys, 208 girls; M_age_ = 16.5) recruited from four public schools in Shanghai, located in the east of China. The data of Sample 1 were subjected to item analysis and exploratory factor analysis.

Sample 2, to whom the formal scale was administered, comprised 542 senior high school students (249 boys, 293 girls; M_age_ = 15.5) recruited from four public schools in Shanghai. The data from Sample 2 were subjected to internal consistency reliability analysis and confirmatory factor analysis.

All the participants were recruited *via* convenience sampling of the adolescent students attending schools, and parents or legal guardians provided written consent for their children to cooperate with our research. This study was approved by the ethics committee of East China Normal University (HR 095 in 2019). Written informed consent was obtained from all participants and their parents in China.

### Items and scoring method

The Health Behavior Scale for senior high school students includes 54 items. Students are required to answer according to their actual situation. Each item is scored on a five-point scale from 1 to 5, each number on the scale corresponding to “completely disagree,” “basically disagree,” “somewhat agree,” “basically agree,” and “completely agree,” respectively. In this study, all items were scored normally except the fifth and eighth items which used reverse scoring; these were summed up *via* coding.

### Statistical analysis

SPSS 26.0 and AMOS 24.0 were used to analyze the data. The first step was a normality test. Means and standard deviations were calculated for all variables. The second step was item analysis, which was used to test the appropriateness or reliability of individual items in the scale. The results of item analysis (i.e., critical ratio and homogeneity testing) were used as a basis to filter or delete items. The third step was exploratory factor analysis (EFA), which is a common method used for scale development and includes reliability tests and validity tests. The fourth step was confirmatory factor analysis (CFA), enabling the structural equation model, discriminant validity, and convergent validity of the scale to be tested. The steps and judgments criteria taken for psychometric assessments have been presented in the Supplementary materials.

## Results

The response rate of Sample 1 was 100%, and the effective rate was 97%. For Sample 2, the response rate was 99% and the effective rate was 96%. All data were strictly screened to remove extreme responding (answering each question with the same answer, i.e., only 1 or 5) and pattern responding (following a certain artificial rule, such as “5, 4, 3, 2, 1, 5, 4, 3, 2, 1,” or “1, 1, 1, 2, 2, 2, 3, 3, 3, 4, 4, 4, 5, 5, 5”). There were no missing data in either sample and no violations of normality in total score distributions were evident. Moreover, the skewness and kurtosis values for the items were within acceptable limits across the samples.

### Item analysis

Item analysis was conducted on the preliminary 54 items. According to the results, Q8 (“I eat fast.”) had four indicators [critical ratio > 3.0, item-total correlation ≥0.4 (Minglong, 2010a), communality ≥0.2 (Yong and Pearce, 2013), and factor loading ≥0.45 (Minglong, 2010b)] below the judgment criterion. Thus, this item (Q8) was deleted (see Table 1).

**Table 1:** Item analysis summary.

Items	Critical ratio	Item-total correlation	Commonalities	Factor loading	Substandard index	Note
**Q1**	14.471	0.675^**^	0.462	0.68	0	retain
Q2	15.197	0.647^**^	0.397	0.63	0	retain
Q3	14.611	0.667^**^	0.44	0.663	0	retain
Q4	18.284	0.674^**^	0.431	0.657	0	retain
Q5	16.484	0.677^**^	0.449	0.67	0	retain
Q6	20.324	0.719^**^	0.485	0.696	0	retain
Q7	16.588	0.634^**^	0.369	0.607	0	retain
Q8	2.372	0.028	0.000	0.020	4	delete
Q9	19.498	0.694^**^	0.469	0.685	0	retain
Q10	14.354	0.678^**^	0.483	0.695	0	retain
Q11	13.769	0.673^**^	0.485	0.696	0	retain
Q12	17.451	0.642^**^	0.391	0.625	0	retain
Q13	17.382	0.622^**^	0.364	0.603	0	retain
Q14	15.877	0.703^**^	0.499	0.706	0	retain
Q15	20.901	0.780^**^	0.606	0.779	0	retain
Q16	15.048	0.645^**^	0.417	0.646	0	retain
Q17	13.71	0.636^**^	0.414	0.644	0	retain
Q18	18.059	0.647^**^	0.403	0.635	0	retain
Q19	19.124	0.747^**^	0.566	0.752	0	retain
Q20	18.998	0.723^**^	0.538	0.733	0	retain
Q21	15.765	0.704^**^	0.523	0.723	0	retain
Q22	13.434	0.678^**^	0.493	0.702	0	retain
Q23	17.915	0.729^**^	0.548	0.74	0	retain
Q24	16.836	0.695^**^	0.482	0.694	0	retain
Q25	16.053	0.705^**^	0.522	0.723	0	retain
Q26	14.57	0.708^**^	0.534	0.731	0	retain
Q27	15.845	0.718^**^	0.551	0.742	0	retain
Q28	19.844	0.767^**^	0.601	0.775	0	retain
Q29	19.354	0.732^**^	0.554	0.744	0	retain
Q30	18.972	0.692^**^	0.47	0.686	0	retain
Q31	21.143	0.739^**^	0.538	0.734	0	retain
Q32	19.58	0.698^**^	0.477	0.691	0	retain
Q33	20.848	0.722^**^	0.504	0.71	0	retain
Q34	22.557	0.730^**^	0.515	0.718	0	retain
Q35	21.568	0.751^**^	0.57	0.755	0	retain
Q36	24.894	0.800^**^	0.623	0.79	0	retain
Q37	20.409	0.761^**^	0.596	0.772	0	retain
Q38	16.939	0.699^**^	0.508	0.712	0	retain
Q39	18.083	0.733^**^	0.556	0.745	0	retain
Q40	19.92	0.780^**^	0.616	0.785	0	retain
Q41	16.93	0.739^**^	0.573	0.757	0	retain
Q42	19.087	0.731^**^	0.541	0.735	0	retain
Q43	18.462	0.725^**^	0.548	0.74	0	retain
Q44	14.275	0.650^**^	0.437	0.661	0	retain
Q45	21.102	0.758^**^	0.583	0.763	0	retain
Q46	23.211	0.713^**^	0.48	0.693	0	retain
Q47	21.854	0.735^**^	0.531	0.729	0	retain
Q48	16.096	0.646^**^	0.434	0.659	0	retain
Q49	19.611	0.664^**^	0.422	0.649	0	retain
Q50	16.354	0.673^**^	0.474	0.689	0	retain
Q51	17.8	0.691^**^	0.477	0.69	0	retain
Q52	18.835	0.673^**^	0.452	0.673	0	retain
Q53	20.228	0.656^**^	0.4	0.632	0	retain
Q54	18.6	0.704^**^	0.502	0.709	0	retain
Judgment criterion	≥3.00	≥0.400	≥0.200	≥0.450		

**p < 0.01.

### Exploratory factor analysis

Validity was tested *via* Kaiser-Meyer-Olkin (KMO) and Bartlett’s test of sphericity. The KMO value was 0.973, greater than 0.60 (Beavers et al., 2013), while Bartlett’s test of sphericity showed high significance (***χ***^***2***^ = 230, 05786.12, ***df*** = 1,378, ***p*** < 0.01), indicating the existence of common factors among variables which are very suitable for factor analysis.

In exploratory factor analysis (EFA), items with factor loads below 0.45 were removed in the next rotation; only one item was deleted at a time. Reanalysis was conducted with new data after each deletion and then the next EFA was conducted. Items with low factor loads were the first to be deleted (such as less than 0.45), followed by the item with the largest cross-factor load. Finally, items with less than three questions in the factors were deleted. A 23-item solution was achieved in 15 iterations and yielded a KMO measure of sampling adequacy of 0.957, and a good Bartlett’s test of sphericity (***χ***^***2***^ = 9105.670, and ***p*** < 0.00). From the results of the principal component analysis using varimax rotation, four common factors with 23 items were extracted (Table 2). The four-factor structure was maintained perfectly regarding the item inclusion criteria, with sufficient loadings and no cross-loading. Commonalities of the variables ranged between 0.58 and 0.84. The first factor included 10 items and accounted for 26.6% of the variance; it was labeled “mastering and applying healthy behavior knowledge.” The second factor included five items and accounted for 17.8% of the variance; it was labeled “exercise awareness and habits.” The third factor included four items and accounted for 14.0% of the variance; it was labeled “environment adaptation.” The fourth component included four items and accounted for 12.2% of the variance; it was labeled “emotional regulation.” The details for each factor as well as the 23 items are shown in Table 2. The Cronbach’s alpha values for each factor as well as for the overall scale were high, namely, 0.937 for Factor 1, 0.907 for Factor 2, 0.863 for Factor 3, 0.874 for Factor 4, and 0.958 for the overall scale.

**Table 2:** Results of exploratory factor analysis.

Item	Component				Commonalities
	Factor 1	Factor 2	Factor 3	Factor 4	
I will actively try my best to prevent all kinds of diseases.	0.796				0.743
I have the awareness and ability regarding security precautions.	0.789				0.749
I understand the harm, routes of transmission, and preventive measures of infectious disease.	0.784				0.723
I have a comprehensive grasp of methods of self-protection and mutual protection in exercise.	0.716				0.717
I never litter and I can sort garbage.	0.708				0.644
I understand the harm of malnutrition to health.	0.680				0.632
I know the characteristics and changing rules of psychological development during puberty.	0.656				0.623
I have good personal and public health habits.	0.626				0.580
I understand that different intensities of exercise require different nutritional needs.	0.611				0.606
I have a good sense of health and pay attention to developing a healthy and civilized lifestyle.	0.524				0.639
Even if there is no physical examination, I will still stick to physical exercise.		0.840			0.794
I have good physical exercise habits.		0.784			0.773
I can actively participate in or organize sports competitions in my class.		0.777			0.716
I know that physical exercise produces more positive emotions than negative emotions.		0.746			0.720
I can keep exercising for my favorite sports.		0.743			0.701
I can quickly adapt to a new learning and living environment.			0.805		0.796
I have good social communication abilities.			0.778		0.774
I will take the initiative to ask my classmates to do physical exercise together in a new class.			0.712		0.720
I know that a harmonious combination of competition and cooperation will make me progress faster.			0.564		0.620
I can distinguish between positive and negative emotions.				0.761	0.844
I know depression is a negative emotion.				0.707	0.703
I have a positive, optimistic, and cheerful attitude towards life.				0.598	0.723
I understand the harm of unhealthy emotions to health.				0.518	0.695
Eigenvalue	6.115	4.105	3.223	2.811	—
Explanatory variance	26.6%	17.8%	14.0%	12.2%	—
Cumulative % of explanatory variance	26.6%	44.4%	58.4%	70.7%	—

Factor 1: mastering and applying healthy behavior knowledge; Factor 2: exercise awareness and habits; Factor 3: environment adaptation; Factor 4: emotional regulation.

### Confirmatory factor analysis

#### Structural equation models

**Figure 1:**
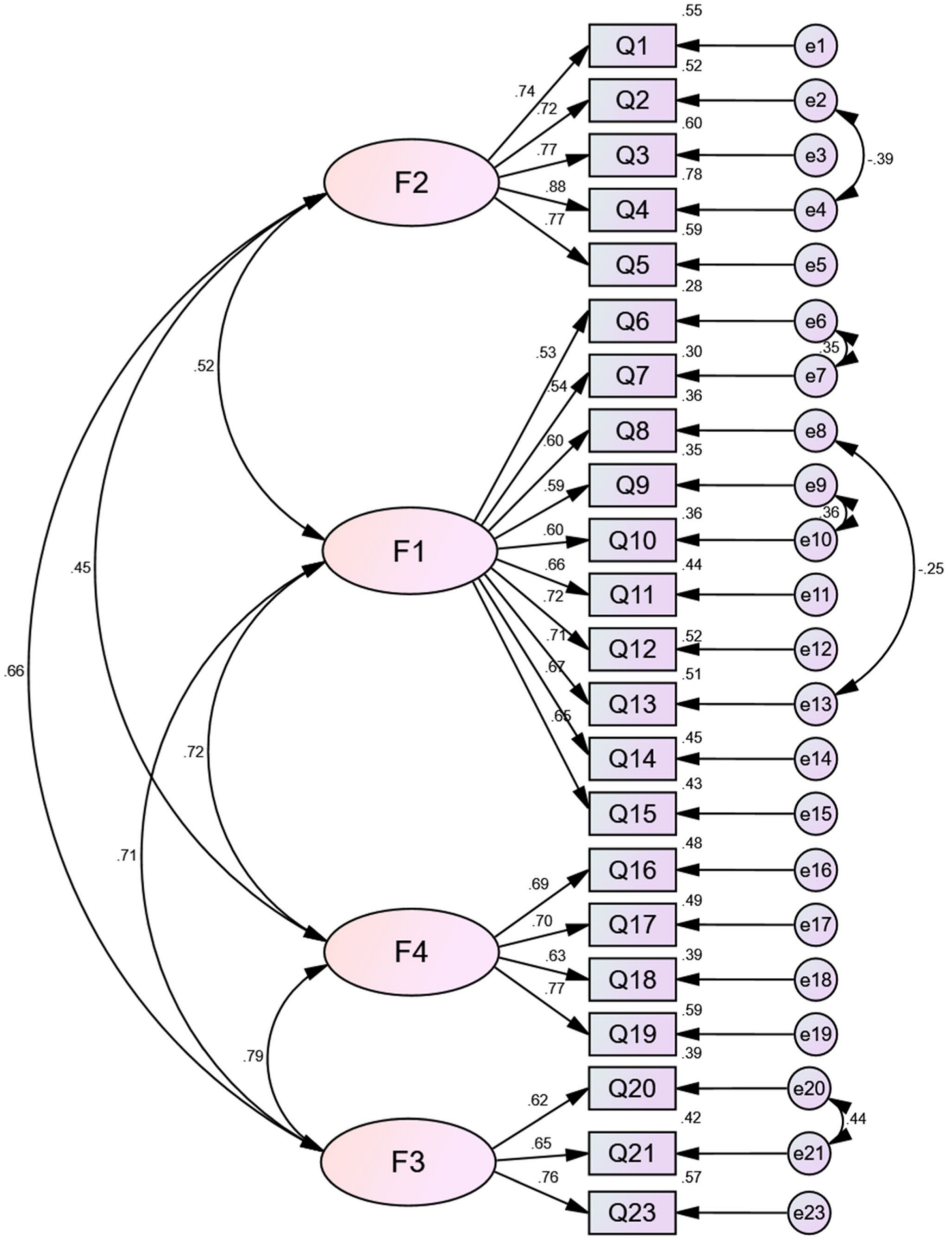
Structural equation model of health behavior.

The 23-item four-factor EFA solution was then modeled using the AMOS program. A Maximum Likelihood CFA procedure executed on Sample 2 (N = 542) did not yield satisfactory fit indices. Therefore, using some of the suggested modification indices to reduce cross-loading, one item was removed (Q22), and to account for some within-factor non-zero correlations between unobserved error variances, some correlation arcs were added to the unobserved error measures. The final four-factor health behavior structure model had 22 items with the following fit statistics: RMSEA = 0.06 (<0.08; Browne and Cudeck, 1992; Hu and Bentler, 1999), CMIN/DF = 2.92 (<5; Bentler and Bonett, 1980; Marsh et al., 1988; Zhong Lin et al., 2004), RMR = 0.03 (<0.05; Minglong, 2010c), CFI = 0.93 (≥ 0.90), GFI = 0.91 (≥ 0.90), TLI = 0.92 (≥0.90; Mcdonald and Ho, 2002; Gundy et al., 2012). The model fitting index is shown in Table 3. These indices represent a good fit of the model based on the reported criteria. Since the CFA led to further elimination of items, an additional EFA was performed on Sample 1 to validate the final 22-item Health Behavior Scale (Figure 1). This EFA perfectly replicated the factor structure of the CFA. The solution explained 71% of the cumulative variance. Besides this, the factor loadings ranged between 0.53 and 0.88 and were significant, indicating a good relationship between the observed variable and latent variable (Ruan et al., 2019).

**Table 3:** Goodness-of-fit of the health behavior model.

	CMIN/DF	RMR	GFI	TLI	CFI	RMSEA
Initial structural model	4.07	0.04	0.86	0.87	0.88	0.08
Modified structural model	2.92	0.03	0.93	0.92	0.91	0.06
Recommended value	1–3	< 0.05	≥ 0.90	≥ 0.90	≥ 0.90	< 0.08

Initial structural model: the structural model before deleting Q22. Modified structural model: the structural model after deleting Q22, and modified model according to the modification indices.

In summary, several rounds of exploratory and confirmatory factor analyses yielded a 22-item scale. The structure that emerged in our data comprised four distinct factors: the first, “mastering and applying healthy behavior knowledge” (10 items), included disease prevention and control, safety consciousness, basic health knowledge, rational nutrition, and a healthy lifestyle. The second, “exercise awareness and habits” (5 items), included exercise habits, exercise persistence, and emotions associated with the exercise of senior high school students. The third, “environment adaptation” (3 items), included social community ability, adaptability, and the ability to deal with the relationship between cooperation and competition. The fourth, “emotional regulation” (4 items), included the understanding of emotions and the identification of different emotions.

#### Convergent and discriminant validity

Three methods were used to assess convergent validity: factor loading, average variance extracted (AVE), and composite reliability (CR). The values of AVE and CR can be found in Table 4. The discriminant validity can be evaluated by the square root of AVE, which is shown in Table 5.

**Table 4:** Convergent validity of the Health Behavior Scale.

Path	Factor loading (> 0.5)	AVE (> 0.36)	CR (> 0.7)
Q1←F2	0.745	0.608	0.885
Q2←F2	0.724		
Q3←F2	0.773		
Q4←F2	0.881		
Q5←F2	0.766		
Q8←F1	0.604	0.399	0.868
Q9←F1	0.595		
Q10←F1	0.597		
Q11←F1	0.66		
Q12←F1	0.724		
Q13←F1	0.713		
Q6←F1	0.528		
Q7←F1	0.544		
Q15←F1	0.652		
Q14←F1	0.669		
Q16←F4	0.693	0.490	0.793
Q17←F4	0.703		
Q18←F4	0.628		
Q19←F4	0.768		
Q20←F3	0.621	0.460	0.717
Q21←F3	0.648		
Q23←F3	0.757		

AVE: Average Variance Extracted. CR: Composite Reliability.

**Table 5:** Discriminant validity of the Health Behavior Scale.

	F1	F2	F3	F4
F1	0.399			
F2	0.142***	0.608		
F3	0.135***	0.271***	0.460	
F4	0.12***	0.163***	0.199***	0.490
The square root of AVE	0.632	0.780	0.678	0.700

Significance level: *** p < 0.001.

#### Test–retest reliability

Test–retest reliability was calculated for the health behavior scale using a sample of 60 senior high school students who completed the health behavior scale a second time after 2 weeks. The correlation coefficients for test–retest reliability ranged from 0.79 to 0.83. The intraclass correlation coefficients of the four factors were 0.83, 0.82, 0.79, 0.80, and 0.83 for the overall scale.

## Discussion

### Result interpretation

We designed and validated an instrument to assess the health behaviors of senior high school students in Shanghai, China. To the best of our knowledge, this is one of the earliest studies to have developed and verified the Health Behavior Scale based on the Physical Education and Health Curriculum Standard (2017 edition).

Through factor analysis, we found that the scale has good reliability and validity. The EFA results (see Table 2) showed that the explanation rate of the cumulative variance after rotation was 70.7%, which was greater than 50%, indicating that the amount of information of the item can be effectively extracted. Meanwhile, the Cronbach’s alpha values of each of the factors was close to or over 0.9, and the Cronbach’s alpha value of the scale was 0.958, suggesting good reliability and high internal consistency for each factor and the scale as a whole (Gliem and Gliem, 2003). The Health Behavior Scale comprises four distinctive dimensions: mastering and applying healthy behavior knowledge, exercise awareness and habits, environment adaptation, and emotional regulation. This is consistent with the point of view put forward by the Physical Education and Health Curriculum Standard (2017 edition).

Regarding the goodness-of-fit of the health behavior model (see Table 3), the indices represent a relatively good fit of the model based on the reported criteria (Hu and Bentler, 1999). Bentler and Bonnet suggest the CMIN/DF as an appropriate measure of model fit, which should not exceed 5 (Bentler and Bonett, 1980). If the CMIN/DF is between 1 and 3, it means that the model fits well, while if the value is less than 5, it means the value in an acceptable range (Marsh et al., 1988). Besides this, the factor loadings ranged between 0.53 and 0.88 and were significant, indicating a good relationship between the observed variable and latent variable. Discriminant validity, along with convergent validity, is a subtype of construct validity (Cronbach and Meehl, 1955).

Discriminant validity shows that two measures that are not supposed to be related are unrelated. Convergent validity takes two measures that are supposed to be measuring the same construct and shows that they are related. In short, discriminant validity focuses on inter-factor correlations, while convergent validity focuses on inter-item correlations. The discriminant validity was established if the inter-test correlation was low, and the convergent validity was established if the inter-item correlation was high. The results of convergent validity (see Table 4) and discriminant validity (see Table 5) suggested that the construct validity of the scale was acceptable. Discriminant validity can be evaluated by comparing the correlation between the same constructs and the square root of AVE for any two constructs (Abdullah et al., 2013; Purnomo, 2017). The absolute values of the correlation coefficients were all less than 0.5, and less than the square root of the corresponding AVE, which indicated that there was a correlation between the latent variables and a certain degree of discrimination between them. This demonstrated that the discriminant validity of the scale was ideal. As can be seen in Table 5, the scale has adequate discriminant validity.

The general rule suggested for AVE is that it should be equal to or greater than 0.50, indicating adequate convergence (Richard and Youjae, 1988; Abdullah et al., 2013). According to Fornell and Larcker (1981), AVE should exceed 0.5 under ideal conditions, while 0.36–0.5 is acceptable (Zhang and Zheng, 2021). Coefficients of 0.5 for standardized factor loading, 0.7 for CR, and 0.36 for AVE are adequate limits for these measures. Hence, all items for convergent validity were met.

The intraclass correlation coefficient (ICC; Bartko, 1966) is one of the reliability coefficient indexes to measure test–retest reliability. The value of ICC lies between 0 and 1, with 0 indicating incredible. The value of ICC lies between 0 and 1, with 0 indicating not credible and 1 indicating completely credible. The ICC values ranging below 0.40 indicate poor reliability, values from 0.40 to 0.59 indicate fair reliability, values from 0.60 to 0.74 indicate good reliability, and values from 0.75 to 1.00 indicate excellent reliability (Portney and Watkins, 1993). The result of ICC demonstrated that the test–retest of the scale was ideal. Due to cultural differences, the concept of health behavior is different from the international interpretation of health behavior. According to the curriculum standard (2017 edition), health behavior is an aspect of the core literacy of the physical education and health discipline. Health behavior is a comprehensive manifestation of improving physical and mental health and actively adapting to the external environment, and it is the key to raising health awareness, improving health status, and gradually forming a healthy and civilized lifestyle. Health behavior includes developing a good exercise routine, rational diet, regular rest, good hygiene, controlling one’s weight, avoiding bad hobbies, preventing exercise injuries and diseases, eliminating exercise fatigue, maintaining a good state of mind, and having the ability to adapt to the natural and social environment. In summary, health behavior refers to all health-related behaviors, not only including behaviors at the conscious level but also at the behavioral level. Internationally, health behaviors, sometimes called health-related behaviors, are considered to be actions taken by individuals that affect health or mortality. These actions may be intentional or unintentional and can promote or detract from the health of the individual or others (Short and Mollborn, 2015). Smoking, drinking, diet, physical activity, sleep, and drug abuse are all indicators of health behaviors. The international definition of health behavior interprets it at a specific behavior level, while the Chinese definition interprets it on an abstract level of consciousness containing more comprehensive content.

Different concepts lead to different measurement methods. International health behavior measurement research includes consideration of the time, frequency, and duration to perform the behaviors, such as the HBSC international research protocol, as well as the GSHS core questionnaire and expanded questionnaire (Health Behaviour in School-aged Children, 2017; World Health Organization, 2021). Compared with international research, the classification and measurement of Chinese health behaviors are not comprehensive. For example, emotional regulation of health behavior includes the cognition of emotions, recognition of different emotions, and the method of emotion regulation in the curriculum standard. It is not an assessment of the extent to which adolescents experience either depression or anxiety, but explores whether adolescents know that depression is a negative emotion and that negative emotions can impact health.

Cultural differences may partially explain the discrepancy, and another reason may be that China began its research on health behavior at a relatively late stage compared to international health behavior research. The measurement of various indicators of health behavior has not been combined with the time and frequency of specific behavior that occurred. The time and frequency of high school students’ health behaviors are not mentioned in the curriculum standard (2017 edition), and nor are the specific indicators of health behaviors. Because health behavior is a macro concept that includes a variety of different indicators, indicators are constantly changing over time. For example, sedentary behavior, which might not have existed in the last century, has become very common in this era. Another reason may be discipline integration. In China, different health behaviors used to be classified in different disciplines. Physical education focuses on the prevention of and how to deal with sports injuries; medicine mainly concerns the treatment and prevention of diseases of the internal medical system, the surgical system, and infectious diseases, such as diabetes, hypertension, cancer, and acquired immunodeficiency syndrome; psychology mainly focuses on mental health problems or mental illness, e.g., anxiety or depression. A growing body of research suggests that exercise during the post-operative rehabilitation period is of importance. Exercise can also promote healthy behaviors and alleviate mental illness. The Health China 2030 plan, released by the State Council of China in 2016, clearly proposes to strengthen the integration of physical and medical interventions and non-medical health interventions. China is moving towards multi-disciplinary integration. Therefore, investigating health behavior in physical education and health involves considering the relevant knowledge from different disciplines, aiming at cultivating health consciousness and promoting the healthy behavior of adolescents.

In summary, this is the first preliminary validation to assess the health behaviors of senior high school students. We found the Health Behavior Scale to be reliable as a valid preliminary measure of health behaviors in this sample. It can also provide a good assessment of the health behaviors of late adolescents and serve as a basis for physical education teachers to better cultivate the core literacy of physical education subjects. Moreover, the scale is based on the national curriculum standards.

### Limitations and areas of future research

There are several study limitations to address. First, although we had an adequate sample size, as confirmed by the KMO and Bartlett’s test results, our sample was recruited from a single city, Shanghai. This may limit its generalizability to the national scale, particularly in the western rural area. And the definition of health behavior is based on the Chinese policy environment and cultural background. This could limit the applicability of this scale to other cultures and countries. Second, primary and middle school students and senior three students were excluded from this study. Future research is required that broadens the assessment of the scale’s validity to early adolescents and youth. Third, the health behavior scale showed consistent factor structure, high internal consistency, good validity, and high test–retest reliability. However, we did not assess criterion validity. Fourth, we only discussed and analyzed the interview results without coding the interview results and using a qualitative research approach to analyze them, which is a limitation. Seven physical education subject specialists and four senior high school physical education teachers only evaluated the content of the scale and did not fill in the expert questionnaire. There was no formal expert questionnaire data, so CVI and CVR could not be calculated. Fifth, we only measured the adolescent comprehension of health behavior (Q18: “I know depression is a negative emotion”) and did not measure the actual health behavior problems experienced by adolescents. We did not evaluate the extent of depression and anxiety in adolescents. Future research will draw on international studies to investigate the health behavior of Chinese adolescents and research the time, frequency, and duration of health behaviors. At the same time, a longitudinal study will deeply analyze Chinese adolescents’ health trends.

Although the scale has various limitations, it was developed to measure the health behaviors of senior high school students and involved the largest sample among studies conducted in China on this topic to date. Our scale could contribute to a further understanding of the situation among senior high school students.

## Conclusion

Previous research on health behaviors focused on adults and the elderly, and few previous studies have comprehensively assessed trends in health behaviors among Chinese late adolescents. Using 1,068 senior high school students recruited from public schools in Shanghai, China, we developed a Health Behavior Scale for senior high school students. Our analysis identified four factors with 23 items: mastering and applying healthy behavior knowledge, exercise awareness habits, environment adaptation, and emotional regulation. Our study suggests that the Health Behavior Scale is a valid instrument for assessing senior high school students’ health behaviors. This scale contributes to the Physical education teachers’ better understanding of the level of health behavior of adolescents, improves the quality of teaching, and then increases adolescents’ health literacy, promoting adolescents’ health. These findings have important implications for enhancing the well-being of late adolescents.

## Data availability statement

The original contributions presented in the study are included in the article/Supplementary materials; further inquiries can be directed to the corresponding author.

## Ethics statement

The studies involving human participants were reviewed and approved by the ethics committee of East China Normal University (HR 095 in 2019). Written informed consent to participate in this study was provided by the participants’ legal guardian/next of kin. Written informed consent was obtained from the minor(s)’ legal guardian/next of kin for the publication of any potentially identifiable images or data included in this article.

## Author contributions

QQ, SD, and JY contributed to the conception and design of the study. SD and JY were involved in implementing the study and data collection. QQ undertook data analysis. QQ and SD wrote the first draft of the manuscript. QQ Polished and revised the draft. All authors contributed to the article and approved the submitted version.

## Funding

This research was funded by East China Normal University’s 2019 outstanding doctoral students’ academic ability improvement project, grant number YBNLTS2019-057.

## Conflict of interest

The authors declare that the research was conducted in the absence of any commercial or financial relationships that could be construed as a potential conflict of interest.

## Publisher’s note

All claims expressed in this article are solely those of the authors and do not necessarily represent those of their affiliated organizations, or those of the publisher, the editors and the reviewers. Any product that may be evaluated in this article, or claim that may be made by its manufacturer, is not guaranteed or endorsed by the publisher.
