# Supplementary material for: Health behaviors of late adolescents in China: Scale development and preliminary validation
Source: Front Psychol. 2022 Nov 11;13:1004364. doi: 10.3389/fpsyg.2022.1004364 (PMC9692111; doi:10.3389/fpsyg.2022.1004364)
Supplement: Supplementary file 1 [file Data_Sheet_1.docx]

**Appendix 1:**

**The interview discussion guide**

Open and closed questions applied during the interviews to physical education subject specialists (N = 5) and physical education teachers (N = 5) from Shanghai, to develop the senior high school students’ health behaviors scale.

| **Closed questions** | **Open questions** |
| --- | --- |
| - Interview Time： - Interview location： - Name： - Sex： - Age： - Education background： - Profession title： - Academic Ranking： - Teaching experience： | - The General Senior High School Physical Education and Health Curriculum Standards (2017 edition) health behavior into four aspects, Exercise awareness and habits, mastering and applying healthy behavior knowledge, emotional regulation, and environment adaptation. Do you think it is reasonable? What contents need to be added or deleted? - Do you think the content about the four aspects of senior high school students health behaviors in the curriculum standard is reasonable? Is there anything that needs to be added or deleted? - In terms of health behavior, what aspects of knowledge do current Chinese senior high school students need to enhance compared with international adolescents? |

**Appendix2:**

**The Late Adolescents' Health Behavior Scale items pool**

Distinguished Professor:

The following are the items pool of the Late Adolescents' Health Behavior Scale.Please tick each item select the most appropriate option and tick "√" on the degree codes 1, 2, 3, 4, 5. 1 means "very inappropriate", 5 means "very suitable". Each of your suggestions is very important to this study. Thank you for your careful guidance and efforts support, thank you!

| Item | | very inappropriate | inappropriate | not clear | suitable | very suitable |
| --- | --- | --- | --- | --- | --- | --- |
| 1 | I understand the importance of physical exercise to physical and mental health. | 1 | 2 | 3 | 4 | 5 |
| 2 | I can actively participate in or organize sports competitions in my class. | 1 | 2 | 3 | 4 | 5 |
| 3 | I know that physical exercise produces more positive emotions than negative emotions. | 1 | 2 | 3 | 4 | 5 |
| 4 | I can develop an exercise plan that suits my health and stick to it | 1 | 2 | 3 | 4 | 5 |
| 5 | Even if there is no physical examination, I will still stick to physical exercise. | 1 | 2 | 3 | 4 | 5 |
| 6 | I can take part in exercise against all odds. | 1 | 2 | 3 | 4 | 5 |
| 7 | I can keep exercising for my favorite sports. | 1 | 2 | 3 | 4 | 5 |
| 8 | I have good physical exercise habits. | 1 | 2 | 3 | 4 | 5 |
| 9 | I have a fixed exercise time every week. | 1 | 2 | 3 | 4 | 5 |
| 10 | I eat fast. | 1 | 2 | 3 | 4 | 5 |
| 11 | I have a regular place to exercise. | 1 | 2 | 3 | 4 | 5 |
| 12 | I have good eating habits. | 1 | 2 | 3 | 4 | 5 |
| 13 | I know the impact of a balanced diet on health, and can guide myself and my family to eat reasonably. | 1 | 2 | 3 | 4 | 5 |
| 14 | I have good personal and public health habits. | 1 | 2 | 3 | 4 | 5 |
| 15 | I never litter and I can sort garbage . | 1 | 2 | 3 | 4 | 5 |
| 16 | I have good work and rest habits. | 1 | 2 | 3 | 4 | 5 |
| 17 | I can work and rest on time to ensure enough sleep. | 1 | 2 | 3 | 4 | 5 |
| 18 | I understand that physical exercise can control weight. | 1 | 2 | 3 | 4 | 5 |
| 19 | I can explain the relationship between reasonable diet and weight control based on my own situation. | 1 | 2 | 3 | 4 | 5 |
| 20 | I can say more than 2 effective ways to refuse smoking. | 1 | 2 | 3 | 4 | 5 |
| 21 | I can consciously resist bad information on the Internet. | 1 | 2 | 3 | 4 | 5 |
| 22 | My main purpose of going online is to search for and learn related information. | 1 | 2 | 3 | 4 | 5 |
| 23 | I understand the prevention and treatment of common physiological problems. | 1 | 2 | 3 | 4 | 5 |
| 24 | I have a basic knowledge of adolescent health care. | 1 | 2 | 3 | 4 | 5 |
| 25 | I know the characteristics and changing rules of psychological development during puberty. | 1 | 2 | 3 | 4 | 5 |
| 26 | I understand the harm of malnutrition to health. | 1 | 2 | 3 | 4 | 5 |
| 27 | I understand that three without products should be rejected when shopping. | 1 | 2 | 3 | 4 | 5 |
| 28 | I understand that different intensities of exercise have different nutritional needs. | 1 | 2 | 3 | 4 | 5 |
| 29 | I understand that outdoor sports can prevent myopia. | 1 | 2 | 3 | 4 | 5 |
| 30 | I understand the harm, routes of transmission, and preventive measures of infectious disease. | 1 | 2 | 3 | 4 | 5 |
| 31 | I will actively try my best to prevent all kinds of diseases. | 1 | 2 | 3 | 4 | 5 |
| 32 | I know that physical exercise needs to be done in the right environment. | 1 | 2 | 3 | 4 | 5 |
| 33 | I understand that physical exercise should be avoided in environments that are not conducive to health (such as haze days). | 1 | 2 | 3 | 4 | 5 |
| 34 | I have the awareness and ability regarding security precautions. | 1 | 2 | 3 | 4 | 5 |
| 35 | I have a comprehensive grasp of methods of self-protection and mutual protection in exercise. | 1 | 2 | 3 | 4 | 5 |
| 36 | I basically master the knowledge and methods to actively avoid danger when natural disasters or emergencies occur. | 1 | 2 | 3 | 4 | 5 |
| 37 | When danger comes, I will lead everyone to take the initiative to avoid danger. | 1 | 2 | 3 | 4 | 5 |
| 38 | I have the knowledge and methods to eliminate sports fatigue. | 1 | 2 | 3 | 4 | 5 |
| 39 | I will relax every time I finish exercising. | 1 | 2 | 3 | 4 | 5 |
| 40 | I master the treatment and first aid methods of common sports injuries and sudden injuries. | 1 | 2 | 3 | 4 | 5 |
| 41 | I can deal with sports injuries that are common in sports activities by myself. | 1 | 2 | 3 | 4 | 5 |
| 42 | I know that I should arrange an appropriate amount of exercise during physical exercise. | 1 | 2 | 3 | 4 | 5 |
| 43 | I often use the knowledge and skills I have learned in physical education class for extracurricular physical exercises. | 1 | 2 | 3 | 4 | 5 |
| 44 | I have a good sense of health and pay attention to developing a healthy and civilized lifestyle. | 1 | 2 | 3 | 4 | 5 |
| 45 | I understand the health risks of sitting too much. | 1 | 2 | 3 | 4 | 5 |
| 46 | I pay a lot of attention to my mental health. | 1 | 2 | 3 | 4 | 5 |
| 47 | I understand the role of physical activity in preventing and removing psychological barriers. | 1 | 2 | 3 | 4 | 5 |
| 48 | I understand the harm of unhealthy emotions to health. | 1 | 2 | 3 | 4 | 5 |
| 49 | I have a positive, optimistic, and cheerful attitude towards life. | 1 | 2 | 3 | 4 | 5 |
| 50 | I can distinguish between positive and negative emotions. | 1 | 2 | 3 | 4 | 5 |
| 51 | I know depression is a negative emotion. | 1 | 2 | 3 | 4 | 5 |
| 52 | I understand the ways of regulating emotions and can adjust my bad emotions in time. | 1 | 2 | 3 | 4 | 5 |
| 53 | When I am in a bad mood, I often take exercise to adjust. | 1 | 2 | 3 | 4 | 5 |
| 54 | I can maintain good emotional stability in sports, study and life. | 1 | 2 | 3 | 4 | 5 |
| 55 | I understand the impact of different environments on exercise. | 1 | 2 | 3 | 4 | 5 |
| 56 | I can still participate in physical exercise even in hot or cold weather. | 1 | 2 | 3 | 4 | 5 |
| 57 | I will take away the rubbish I made when doing outreach training in the wild. | 1 | 2 | 3 | 4 | 5 |
| 58 | I have good social communication abilities. | 1 | 2 | 3 | 4 | 5 |
| 59 | I can quickly adapt to a new learning and living environment. | 1 | 2 | 3 | 4 | 5 |
| 60 | I will take the initiative to ask my classmates to do physical exercise together in a new class. | 1 | 2 | 3 | 4 | 5 |
| 61 | I know that a harmonious combination of competition and cooperation will make me progress faster. | 1 | 2 | 3 | 4 | 5 |

**Appendix 3:**

**Late Adolescents' Health Behavior Scale**

Dear students,

The following lists some statements related to your health. Please read them one by one and determine whether you agree with these statements and the degree of agreement according to your own specific situation. Please mark **“√”** on the number that best matches your question. When answering each question, please follow the criteria below:

1=completely disagree 2=basically disagree 3=somewhat agree

4=basically agree 5=completely agree

The survey results of this questionnaire are only used by scientific research institutes and have nothing to do with your physical education performance. Please be sure to fill in truthfully according to your own ideas. Thank you for your cooperation!

Grade: Class: Gender:

| Item | | completely disagree | basically disagree | somewhat agree | basically agree | completely agree |
| --- | --- | --- | --- | --- | --- | --- |
| 1 | I understand the importance of physical exercise to physical and mental health | 1 | 2 | 3 | 4 | 5 |
| 2 | I can actively participate in or organize sports competitions in my class. | 1 | 2 | 3 | 4 | 5 |
| 3 | I know that physical exercise produces more positive emotions than negative emotions. | 1 | 2 | 3 | 4 | 5 |
| 4 | Even if there is no physical examination, I will still stick to physical exercise. | 1 | 2 | 3 | 4 | 5 |
| 5 | I can keep exercising for my favorite sports. | 1 | 2 | 3 | 4 | 5 |
| 6 | I have good physical exercise habits. | 1 | 2 | 3 | 4 | 5 |
| 7 | I have a fixed exercise time every week | 1 | 2 | 3 | 4 | 5 |
| 8 | I eat fast | 1 | 2 | 3 | 4 | 5 |
| 9 | I know the impact of a balanced diet on health, and can guide myself and my family to eat reasonably | 1 | 2 | 3 | 4 | 5 |
| 10 | I have good personal and public health habits. | 1 | 2 | 3 | 4 | 5 |
| 11 | I never litter and I can sort garbage . | 1 | 2 | 3 | 4 | 5 |
| 12 | I have good work and rest habits | 1 | 2 | 3 | 4 | 5 |
| 13 | I can work and rest on time to ensure enough sleep | 1 | 2 | 3 | 4 | 5 |
| 14 | I understand that physical exercise can control weight | 1 | 2 | 3 | 4 | 5 |
| 15 | I can explain the relationship between reasonable diet and weight control based on my own situation | 1 | 2 | 3 | 4 | 5 |
| 16 | I can say more than 2 effective ways to refuse smoking | 1 | 2 | 3 | 4 | 5 |
| 17 | I can consciously resist bad information on the Internet | 1 | 2 | 3 | 4 | 5 |
| 18 | My main purpose of going online is to search for and learn related information | 1 | 2 | 3 | 4 | 5 |
| 19 | I understand the prevention and treatment of common physiological problems | 1 | 2 | 3 | 4 | 5 |
| 20 | I know the characteristics and changing rules of psychological development during puberty. | 1 | 2 | 3 | 4 | 5 |
| 21 | I understand the harm of malnutrition to health. | 1 | 2 | 3 | 4 | 5 |
| 22 | I understand that three without products should be rejected when shopping | 1 | 2 | 3 | 4 | 5 |
| 23 | I understand that different intensities of exercise have different nutritional needs. | 1 | 2 | 3 | 4 | 5 |
| 24 | I understand that outdoor sports can prevent myopia | 1 | 2 | 3 | 4 | 5 |
| 25 | I understand the harm, routes of transmission, and preventive measures of infectious disease. | 1 | 2 | 3 | 4 | 5 |
| 26 | I will actively try my best to prevent all kinds of diseases. | 1 | 2 | 3 | 4 | 5 |
| 27 | I have the awareness and ability regarding security precautions. | 1 | 2 | 3 | 4 | 5 |
| 28 | I have a comprehensive grasp of methods of self-protection and mutual protection in exercise. | 1 | 2 | 3 | 4 | 5 |
| 29 | I basically master the knowledge and methods to actively avoid danger when natural disasters or emergencies occur | 1 | 2 | 3 | 4 | 5 |
| 30 | When danger comes, I will lead everyone to take the initiative to avoid danger | 1 | 2 | 3 | 4 | 5 |
| 31 | I have the knowledge and methods to eliminate sports fatigue | 1 | 2 | 3 | 4 | 5 |
| 32 | I will relax every time I finish exercising | 1 | 2 | 3 | 4 | 5 |
| 33 | I master the treatment and first aid methods of common sports injuries and sudden injuries | 1 | 2 | 3 | 4 | 5 |
| 34 | I can deal with sports injuries that are common in sports activities by myself | 1 | 2 | 3 | 4 | 5 |
| 35 | I know that I should arrange an appropriate amount of exercise during physical exercise | 1 | 2 | 3 | 4 | 5 |
| 36 | I often use the knowledge and skills I have learned in physical education class for extracurricular physical exercises | 1 | 2 | 3 | 4 | 5 |
| 37 | I have a good sense of health and pay attention to developing a healthy and civilized lifestyle | 1 | 2 | 3 | 4 | 5 |
| 38 | I understand the health risks of sitting too much | 1 | 2 | 3 | 4 | 5 |
| 39 | I pay a lot of attention to my mental health | 1 | 2 | 3 | 4 | 5 |
| 40 | I understand the role of physical activity in preventing and removing psychological barriers | 1 | 2 | 3 | 4 | 5 |
| 41 | I understand the harm of unhealthy emotions to health. | 1 | 2 | 3 | 4 | 5 |
| 42 | I have a positive, optimistic, and cheerful attitude towards life. | 1 | 2 | 3 | 4 | 5 |
| 43 | I can distinguish between positive and negative emotions. | 1 | 2 | 3 | 4 | 5 |
| 44 | I know depression is a negative emotion. | 1 | 2 | 3 | 4 | 5 |
| 45 | I understand the ways of regulating emotions and can adjust my bad emotions in time | 1 | 2 | 3 | 4 | 5 |
| 46 | When I am in a bad mood, I often take exercise to adjust | 1 | 2 | 3 | 4 | 5 |
| 47 | I can maintain good emotional stability in sports, study and life | 1 | 2 | 3 | 4 | 5 |
| 48 | I understand that physical exercise should be avoided in environments that are not conducive to health (such as haze days) | 1 | 2 | 3 | 4 | 5 |
| 49 | I can still participate in physical exercise even in hot or cold weather | 1 | 2 | 3 | 4 | 5 |
| 50 | I will take away the rubbish I made when doing outreach training in the wild | 1 | 2 | 3 | 4 | 5 |
| 51 | I have good social communication abilities. | 1 | 2 | 3 | 4 | 5 |
| 52 | I can quickly adapt to a new learning and living environment. |  | 2 | 3 | 4 | 5 |
| 53 | I will take the initiative to ask my classmates to do physical exercise together in a new class. | 1 | 2 | 3 | 4 | 5 |
| 54 | I know that a harmonious combination of competition and cooperation will make me progress faster. | 1 | 2 | 3 | 4 | 5 |

**Appendix 4:**

**Late Adolescents' Health Behavior Scale**

Dear students,

The following lists some statements related to your health. Please read them one by one and determine whether you agree with these statements and the degree of agreement according to your own specific situation. Please mark **“√”** on the number that best matches your question. When answering each question, please follow the criteria below:

1=completely disagree 2=basically disagree 3=somewhat agree

4=basically agree 5=completely agree

The survey results of this questionnaire are only used by scientific research institutes and have nothing to do with your physical education performance. Please be sure to fill in truthfully according to your own ideas. Thank you for your cooperation!

Grade: Class: Gender:

| Item | | completely disagree | basically disagree | somewhat agree | basically agree | completely agree |
| --- | --- | --- | --- | --- | --- | --- |
| 1 | I can actively participate in or organize sports competitions in my class. | 1 | 2 | 3 | 4 | 5 |
| 2 | I know that physical exercise produces more positive emotions than negative emotions. | 1 | 2 | 3 | 4 | 5 |
| 3 | I can keep exercising for my favorite sports. | 1 | 2 | 3 | 4 | 5 |
| 4 | I have good physical exercise habits. | 1 | 2 | 3 | 4 | 5 |
| 5 | Even if there is no physical examination, I will still stick to physical exercise. | 1 | 2 | 3 | 4 | 5 |
| 6 | I have good personal and public health habits. |  |  |  |  |  |
| 7 | I never litter and I can sort garbage . | 1 | 2 | 3 | 4 | 5 |
| 8 | I know the characteristics and changing rules of psychological development during puberty. | 1 | 2 | 3 | 4 | 5 |
| 9 | I understand the harm of malnutrition to health. | 1 | 2 | 3 | 4 | 5 |
| 10 | I understand that different intensities of exercise have different nutritional needs. | 1 | 2 | 3 | 4 | 5 |
| 11 | I understand the harm, routes of transmission, and preventive measures of infectious disease. | 1 | 2 | 3 | 4 | 5 |
| 12 | I will actively try my best to prevent all kinds of diseases. | 1 | 2 | 3 | 4 | 5 |
| 13 | I have the awareness and ability regarding security precautions. | 1 | 2 | 3 | 4 | 5 |
| 14 | I have a comprehensive grasp of methods of self-protection and mutual protection in exercise. | 1 | 2 | 3 | 4 | 5 |
| 15 | I have a good sense of health and pay attention to developing a healthy and civilized lifestyle | 1 | 2 | 3 | 4 | 5 |
| 16 | I understand the harm of unhealthy emotions to health. | 1 | 2 | 3 | 4 | 5 |
| 17 | I have a positive, optimistic, and cheerful attitude towards life. | 1 | 2 | 3 | 4 | 5 |
| 18 | I know depression is a negative emotion. | 1 | 2 | 3 | 4 | 5 |
| 19 | I can distinguish between positive and negative emotions. | 1 | 2 | 3 | 4 | 5 |
| 20 | I have good social communication abilities. | 1 | 2 | 3 | 4 | 5 |
| 21 | I can quickly adapt to a new learning and living environment. | 1 | 2 | 3 | 4 | 5 |
| 22 | I will take the initiative to ask my classmates to do physical exercise together in a new class. | 1 | 2 | 3 | 4 | 5 |
| 23 | I know that a harmonious combination of competition and cooperation will make me progress faster. | 1 | 2 | 3 | 4 | 5 |

**Supplementary material:**

**The steps and judgments criteria have been taken for psychometric assessments:**

The first step was to determine the descriptive statistics. Means and standard deviations were calculated for all normally distributed variables. The second step was item analysis. We explored the differences among participants in each item after high and low groups to test the homogeneity of items. Then we performed the independent-sample t-tests, Pearson correlation analysis, and internal consistency reliability analyses. After the preliminary scale was tested, item analysis, validity testing, and reliability testing should be carried out as the basis for the development of the formal scale. The result of item analysis (i.e., critical ratio and homogeneity testing) could be used as a basis to filter or delete items. The third step was exploratory factor analysis. Exploratory factor analysis is a common method used for scale development, including reliability tests and validity tests. The fourth step was confirmatory factor analysis (CFA). The construct validity was determined, by building the structural equation model and measuring discriminant validity and convergent validity. The model’s overall goodness of fit was assessed using a combination of indices: CMIN/DF, RMR, GFI, CFI, TLI, and RMSEA. Discriminant validity can be evaluated by comparing the inter-factor correlation and the square root of AVE for any two constructs (Abdullah et al., 2013; Purnomo, 2017). Convergent validity can be evaluated by comparing the inter-item correlation, and the value of AVE and CR. The discriminant validity was established if the inter-test correlation was low, and the convergent validity was established if the inter-item correlation was high. During the analysis process, the following criteria were used to determine whether the scale was reasonable.

1. Critical ratio. The critical ratio is a commonly used discriminant index in item analysis. The critical ratio should be above 3.0.
2. Homogeneity test. In item analysis, in addition to the critical ratio, a homogeneity test can also be used. The homogeneity test includes the correlation between the item and the total score of the scale (item-total correlation), the factor loading of the common factor of the item in the scale, or the internal consistency reliability test value.
3. Kaiser-Meyer-Olkin (KMO). Whether the items are suitable for factor analysis can be judged from the value of the Kaiser-Meyer-Olkin statistics. According to Kaiser's point of view, when the value of KMO is above 0.90, it is marvelous; when 0.80-0.90, meritorious; when 0.70-0.80, middling; when 0.60-0.70, mediocre; when 0.50-0.60, miserable; below 0.50, unacceptable (Li et al., 2020; Henry, 1974). In other words, The KMO statistics range from 0 to 1, and the closer to 1, the more suitable for factor analysis, the minimum recommended value is 0.6 (Beavers et al., 2013).
4. Factor loading. Factor loading reflects the importance of the item to the extracted common factor, and the value cannot be less than 0.4 (Minglong, 2010a).
5. Communality. Communality is the variation in the observed variables which are accounted for by a common factor or common variance (Yong and Pearce, 2013).
6. Cronbach’s alpha coefficient. Cronbach’s alpha coefficient is one of the indices used to test the internal consistency of the scale in reliability analysis. When the Cronbach’s alpha coefficient is above 0.9, the reliability of the scale is ideal; when it is between 0.8 and 0.9, the reliability of the scale is very good; when between 0.7 and 0.8, the reliability of the scale is good; when between 0.6 and 0.7, the reliability of the scale is acceptable; when between 0.5 and 0.6, the scale is acceptable but very low; when below 0.5, the reliability of the scale is unacceptable. In other words, Cronbach’s alpha coefficient ranges from 0 to 1, and the closer Cronbach’s alpha coefficient is to 1.0, the greater the internal consistency of the items in the scale (Gliem and Gliem, 2003).
7. Item-total correlation. The value of the item-total correlation is an index used for judging the internal consistency of the item and the remaining items. If the value is less than 0.4, the internal consistency of the item and the remaining items is low (Minglong, 2010b).
8. The Fit indices of the structural equation model: CMIN/DF, RMR, GFI, CFI, TLI, RMSEA.

CMIN/DF (chi-square/degrees-of-freedom). Bentler and Bonnet suggested the CMIN/DF as an appropriate measure of model fit; it should not exceed 5 (Bentler and Bonett, 1980). If the CMIN/DF is between 1 and 3, it means that the model fits well, and if the value is less than 5, it means that the value is in an acceptable range (Zhonglin et al., 2004; Marsh et al., 1988).

RMR (root mean square residual). The smaller the RMR, the better, and the smaller the value, the better the fit of the model. Generally speaking, RMR below 0.05 indicates an acceptably fitting model (Minglong, 2010c).

GFI (goodness of fit index), CFI (comparative fit index), and TLI (Tucker-Lewis index). The values of GFI, CFI, and TLI range from 0 to 1; the closer the value is to 1, the better the reliability of the scale is (Minglong, 2010b). In general, GFI, CFI, and TLI values greater than 0.90 indicates a good model fit (Gundy et al., 2012; Mcdonald and Ho, 2002).

RMSEA (root mean square error of approximation). If the RMSEA is close to 0.06, we can conclude that there is a relatively good fit (Hu and Bentler, 1999). A commonly used rule of thumb is that an RMSEA less than 0.05 indicates a close approximate fit, while values between 0.05 and 0.08 indicate an acceptable fit (Mcdonald and Ho, 2002), and values above 0.10 indicate a poor approximate fit (Browne and Cudeck, 1992; Hu and Bentler, 1999).

1. Composite Reliability (CR). Composite reliability, which represents the overall reliability of a multi-dimensional construct. Composite reliability is another measure of [internal consistency](https://www.statisticshowto.com/internal-consistency/) in scale items. It is recommended that the reliability of a construct is at least 0.70 (Barclay et al., 1995). High composite reliability is a very good indication that all your items constantly measure the same construct. CR generally appears at the same time as AVE.
2. Average variance extracted (AVE). The average extraction variation is a measure of convergent validity and is the degree to which a latent construct explains the variance of its indicators. According to Fornell and Larcker (1981), AVE should exceed 0.5 under ideal conditions, while 0.36–0.5 is acceptable (Zhang and Zheng, 2021; Fornell and Larcker, 1981).
3. The square root of AVE. The absolute values of the correlation coefficients were all less than 0.5, and less than the square root of the corresponding AVE, which indicated that there was a correlation between the latent variables and a certain degree of discrimination between them.

**REFERENCES**

Barclay, D., Thompson, R., and Higgins, C. (1995). The Partial Least Squares (PLS) Approach to Causal Modeling: Personal Computer Use as an Illustration. *Technology Studies, 2*.

Beavers, A. S., Lounsbury, J. W., Richards, J. K., Huck, S. W., Skolits, G. J., Esquivel, S. L. (2013). Practical considerations for using exploratory factor analysis in educational research. *Practical Assessment, Research & Evaluation.* 18, 6.

Bentler, P. M., and Bonett, D. G. (1980). Significance tests and goodness of fit in the analysis of covariance structures. *Psychol. Bull.* 88.

Browne, M. W., and Cudeck, R. (1992). Alternative ways of assessing model fit. *Sociological methods & research.* 21, 230-258. doi:10.1177/0049124192021002005

Fornell, C., and Larcker, D. F. (1981). Structural Equation Models with Unobservable Variables and Measurement Error：Algebra and Statistics. *Journal of Marketing Research.* 18, 382-388.

Gliem, J. A., and Gliem, R. R. Data from: Calculating, interpreting, and reporting Cronbach's alpha reliability coefficient for Likert-type scales. In 2003 Midwest Research to Practice Conference in Adult, Continuing, and Community Education,82-88.

Gundy, C. M., Fayers, P. M., Groenvold, M., Petersen, M. A., Scott, N. W., Sprangers, M. A. et al. (2012). Comparing higher order models for the EORTC QLQ-C30. *Qual. Life Res.* 21, 1607-1617. doi:10.1007/s11136-011-0082-6

Henry, F. K. (1974). An index of factorial simplicity. *Psychometrika.* 39.

Hu, L., and Bentler, P. M. (1999). Cutoff criteria for fit indexes in covariance structure analysis: conventional criteria versus new alternatives. *Structural Equation Modeling.* 6, 1-55. doi:10.1080/10705519909540118

Li, X., Du J, and Long, H. (2020). Understanding the Green Development Behavior and Performance of Industrial Enterprises (GDBP-IE): Scale Development and Validation. *Int J Environ Res Public Health.* 17. doi:10.3390/ijerph17051716

Marsh, H. W., Balla, J. R., and McDonald, R. P. (1988). Goodness-of-fit indexes in confirmatory factor analysis: the effect of sample size. *Psychol. Bull.* 103, 391-410. doi:10.1037/0033-2909.103.3.391

McDonald, R. P., and Ho, M. R. (2002). Principles and practice in reporting structural equation analyses. *Psychological Methods.* 7, 64-82. doi:10.1037//1082-989X.7.1.64

Minglong, W. (2010a). *Questionnaire Statistical Analysis Practice: SPSS Operation and Application.* Chongqing: Chongqing University Press. p. 188-193.

Minglong, W. (2010b). *Questionnaire Statistical Analysis Practice: SPSS Operation and Application.* Chongqing: Chongqing University Press. p. 200-201.

Minglong, W. (2010c). *Structural Equation Modeling: The Operation and Application of AMOS. 2nd Edition.* Chongqing: Chongqing University Press. p.43-45.

Yong, A. G., and Pearce, S. (2013). A beginner's guide to factor analysis: focusing on exploratory factor analysis. *Quant. Meth. Psych.* 9, 79-94. doi:10.20982/tqmp.09.2.p079

Zhang, Z., and Zheng, L. (2021). Consumer community cognition, brand loyalty, and behaviour intentions within online publishing communities: an empirical study of Epubit in China. *Learned publishing.* 34, 116-127. doi:10.1002/leap.1327

ZhongLin, W., Hau, K., and Herb, M. (2004). Structural equation model testing: cutoff criteria for goodness of fit indices and chi-square test. *Acta Psychologica Sinica*. 36, 186-194.
